# The Clinical Applications of Liquid Biopsies in Pediatric Brain Tumors: A Systematic Literature Review

**DOI:** 10.3390/cancers14112683

**Published:** 2022-05-28

**Authors:** Ladina Greuter, Nicole Frank, Raphael Guzman, Jehuda Soleman

**Affiliations:** 1 Department of Neurosurgery, University Hospital of Basel, 4031 Basel, Switzerland; nicolealexandra.frank@usb.ch (N.F.); raphael.guzman@usb.ch (R.G.); jehuda.soleman@usb.ch (J.S.); 2 Department of Neurosurgery and Pediatric Neurosurgery, University Hospital of Basel and Children’s Hospital, 4056 Basel, Switzerland; 3 Faculty of Medicine, University of Basel, 4056 Basel, Switzerland

**Keywords:** liquid biopsy, pediatric brain tumors, pediatric neurosurgery, diffuse midline glioma, medulloblastoma

## Abstract

**Simple Summary:**

Brain tumors are the most common solid cancer in children and are traditionally diagnosed via a tissue biopsy or resection. Liquid biopsy offers the possibility to characterize brain tumors based on their circulating DNA in blood, cerebrospinal fluid or even urine. Moreover, disease progress can be monitored accurately and sometimes even detected before radiographic progression. More trials are needed to standardize the use of liquid biopsy in pediatric brain tumors.

**Abstract:**

Background: Pediatric brain tumors are the most common solid tumor in children. Traditionally, tumor diagnosis and molecular analysis were carried out on tumor tissue harvested either via biopsy or resection. However, liquid biopsy allows analysis of circulating tumor DNA in corporeal fluids such as cerebrospinal fluid or blood. Methods: We performed a systematic review in Pubmed and Embase regarding the role of liquid biopsy in pediatric brain tumors. Results: Nine studies with a total of 570 patients were included. The preferred corporeal fluid for analysis with a relatively high yield of ct-DNA was cerebrospinal fluid (CSF). For high-grade glioma, liquid biopsy can successfully characterize H3K27mutations and predict tumor progression before it is radiographically detected. Moreover, liquid biopsy has the potential to distinguish between pseudo-progression and actual progression. In medulloblastoma, ct-DNA in the CSF can be used as a surrogate marker of measurable residual disease and correlates with response to therapy and progression of the tumor up to three months before radiographic detection. Conclusion: Liquid biopsy is primarily useful in high-grade pediatric brain tumors such as diffuse midline glioma or medulloblastoma. Disease detection and monitoring is feasible for both tumor entities. More trials to standardize its use for pediatric brain tumors are necessary.

## 1. Introduction

Pediatric brain tumors (PBT) are the most common solid tumors in children, with an incidence of 5.7 per 100,000 children, and the leading cause of tumor-related mortality in children [1,2]. The two most common PBTs are gliomas and medulloblastoma (MB). Gliomas, of which a third are classified as high grade, comprise the majority of all PBTs, whereas medulloblastomas are the most common malignant PBT [3,4]. Over the previous years, considerable advances have been made in classifying these tumors on a molecular basis and identifying mutations in specific tumor types [5,6,7]. This development has also been implemented in the new World Health Organization (WHO) classification of central nervous system (CNS) tumors, which highly relies on molecular classification [8]. Pediatric high-grade gliomas (pHGG) are now classified as diffuse midline gliomas (DMG) H3 K27-altered, diffuse hemispheric glioma H3 G34-mutant, and diffuse pHGG H3-wildtype and IDH-wildtype [8]. Similar changes were also applied to medulloblastomas [8]. Traditionally, these molecular markers are identified on tumor DNA harvested from a tissue sample through biopsy or resection; however, over the last few years, new methods in which circulating cell-free DNA or tumor DNA (ct-DNA), can be harvested from other corporeal fluids such as cerebrospinal fluid (CSF), blood, or urine, the so called ‘liquid biome’, gained popularity [9,10,11]. Such procedures are called liquid biopsies as the tumor DNA stems from a corporeal fluid, instead of the solid tumor itself. Liquid biopsies are especially promising for highly eloquent tumors, such as midline gliomas in the brainstem, in which biopsy carries a high risk of postoperative morbidity [10]. Moreover, traditional tumor biopsy carries the risk of sampling error due to tumor heterogeneity, whereas liquid biopsy by measuring ct-DNA represents the entire tumor [9,11]. Hence, liquid biopsy could be a potential new method for minimal invasive tumor diagnosis and allow for a molecular tumor follow-up and disease monitoring [11,12,13,14]. To detect and quantify ct-DNA, highly sensitive analyses such as digital droplet polymerase chain reaction (PCR) or next generation sequencing are used, which allow detection and quantification of mutant tumor alleles on the backdrop of normal cf-DNA [9,11,14,15,16].

So far, the literature on liquid biopsy in pediatric brain tumors is scarce and mainly limited to midline gliomas and medulloblastomas [13,14]. The aim of this systematic review is to give a comprehensive overview of the present knowledge and role of liquid biopsy in pediatric brain tumors and to present a potential future prospective.

## 2. Materials and Methods

**Figure 1:**
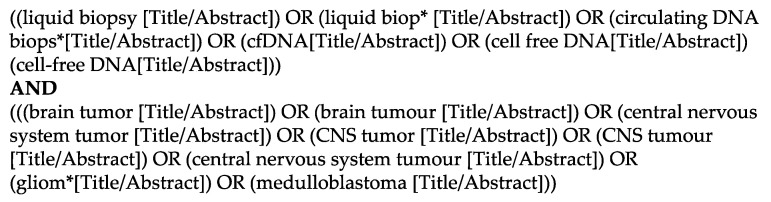
Detailed search string used in Pubmed to identify the relevant studies.

For this systematic review, we searched the databases PubMed and Embase and included articles in English from their induction until the 1st of March 2022. We created a search string including the keywords ‘liquid biopsy’ or ‘cf-DNA’ or ‘ct-DNA’ and ‘brain tumor’ (Figure 1). We included randomized trials, retro- and prospective cohort studies, as well as case series, but excluded reviews and technical reports. We only included studies describing solely PBTs, which we defined as patients <20 years. After removal of duplicates, which was done with the help of the web-based software Rayyan [17], all articles were screened according to their titles by two authors independently (L.G. and N.F.). Thereafter, the remaining articles were assessed according to their abstracts and full-texts by L.G. and N.F. independently and a final list of articles was compiled. In case of a disagreement concerning the in- or exclusion of a study, the senior author (J.S.) took the final decision. This review was performed in accordance with the Preferred Reporting Items for Systematic Reviews and Meta-Analyses (PRISMA) guidelines and registered with the research registry (ID number 7820) [18].

## 3. Results

**Figure 2:**
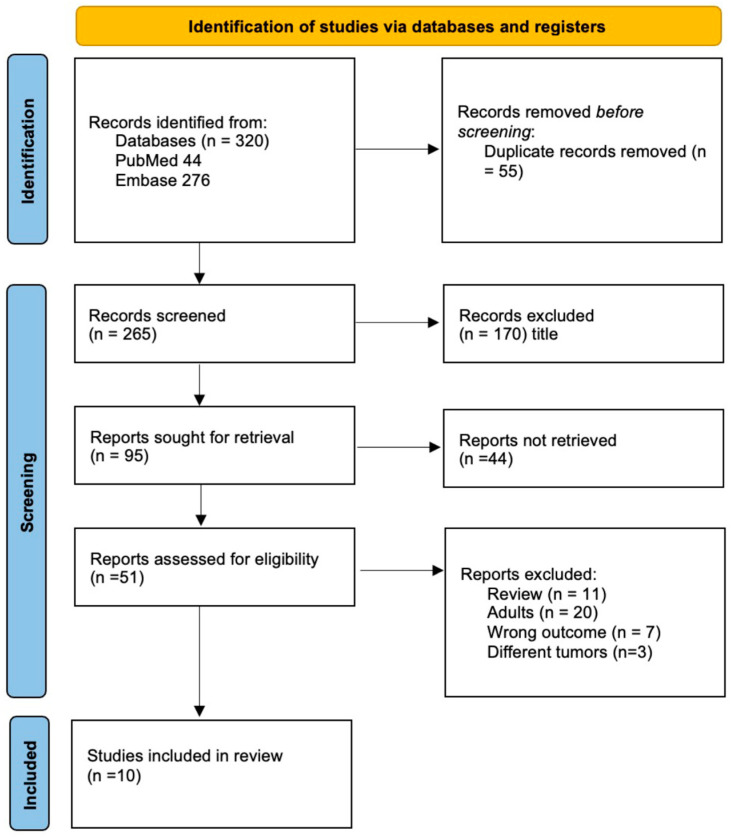
PRISMA flowchart depicting the inclusion process of the studies.

We included nine studies with a total of 570 patients in this systematic review (Figure 2) [13,14,15,16,19,20,21,22,23]. Five studies included only diffuse midline gliomas (127 patients), three studies included medulloblastomas (212 patients) and one study included any CNS tumor (258 patients, Table 1). All studies included were prospective non-randomized trials. Two studies mainly aimed to test if liquid biopsy is feasible to profile pediatric brain tumors [19] and specifically to detect H3 mutations in DMG [21]. One study reported their optimization of ctDNA detection sensitivity in DMG [20]. The majority of studies aimed to investigate whether liquid biopsy allows reliable monitoring of either DMG or MB and allows detection or even prediction of tumor progression by measuring changes in ct-DNA concentrations [13,14,15,16,22,23] (Table 1). Only one technical report describes the successful identification of micro-RNA-21 in five pediatric medulloblastoma patients but, due to its lack of information on clinical data, it was not included in this systematic review [24]. Through our systematic search, no further clinical study on pediatric brain tumors describing liquid biopsy based on RNA were identified.

### 3.1. Analysis of Corporeal Fluids Used

**Figure 3:**
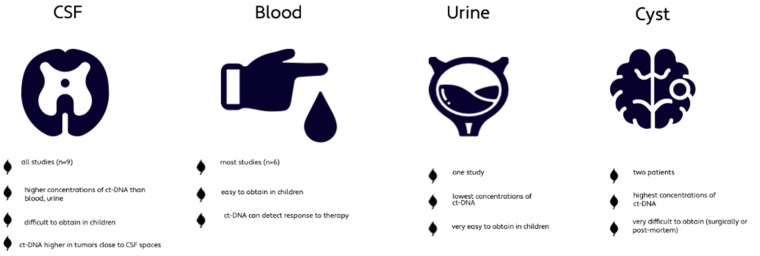
Characteristics of the different corporeal fluids used for liquid biopsy [13,14,15,19].

All studies used CSF as body fluid whereas six studies additionally used blood. Two studies also analyzed tumor cyst fluid and one study investigated urine as well (Table 1). The concentration of ct-DNA is significantly higher in CSF compared to blood or urine of PBT patients [14,15,19]. Cyst fluid, which was only available in two patients (0.3%), showed the highest level of ct-DNA; however, it can only be obtained through surgical procedures or post-mortem [14,15]. Ct-DNA in CSF seemed superior for disease monitoring compared to ct-DNA in plasma [15,23]. Liu et al. detected that ct-DNA levels were increased in MBs located close to the CSF spaces, such as the fourth ventricle, compared to MBs located within the cerebellar hemisphere [13]. The study by Cantor et al. investigating DMG, did not detect any difference in concentration depending on the different locations (brainstem, thalamus, spinal cord) [23], whereas a third study investigated the different concentration of ct-DNA with respect to tumor location and found that ct-DNA is more abundant in the directly adjacent CSF spaces of the tumor [15] (Figure 3). These findings suggest CSF to be the preferred corporeal fluid for liquid biopsies in pediatric brain tumors.

### 3.2. High-Grade Glioma and Diffuse Midline Glioma

Five studies (55.5%) analyzed the efficacy and utility of liquid biopsies in HGG or DMG. Four of the studies included only patients with DMG. One study included in addition to DMG, other high-grade gliomas, whereas one study included various PBTs (258 patients), of which 35 (13.5%) were HGG or DMG [14,15,19,20,21,23]. All these studies investigated their samples for H3K27 mutation. Two studies also detected additional mutations for HGG, such as H3G34 and BRAF (V600E), IDH1, PIK3CA, and TP53 [14,21] (Table 2). In the study by Huang et al., the patient with the H3G34 mutation suffered from a supratentorial glioblastoma and was used as a control for patients in which no H3K27 mutation was expected [21]. Harvesting enough DNA to detect a H3K27 mutation was successful in 67–96% of the collected CSF samples and 33–90% of the collected plasma samples from patients with DMG [14,15,21,23].

Three studies described the feasibility of detecting H3K27 mutations reliably in CSF or serum, with the aim of proving the concept of liquid biopsy [19,20,21]. The other three studies investigated whether liquid biopsy is feasible for monitoring of DMG or HGG and detection, or even prediction, of progression [14,15,23] (Table 2).

One study showed that in 83% of all patients undergoing radiotherapy a significant decrease in ctDNA with H3K27 mutation could be detected in plasma [15]. Moreover, a larger amount of ctDNA was detected in 60% of all patients during disease progression or increase in tumor size [15]. Another study by Cantor et al. showed that a decrease in ct-DNA mutations in CSF correlated significantly with a prolonged progression-free survival (PFS), whereas the number of ct-DNA mutations in plasma did not reach statistical significance [23]. An increase in ct-DNA in plasma was measured before the actual radiographic tumor progression was noticed in 50% and occurred with progression in 18.5%. In the CSF, a ct-DNA spike preceded progression only in 45% [23]. Moreover, in two patients, changes in ct-DNA measuring H3K27M mutations could differentiate between pseudo-progression and pseudo-response after bevacizumab treatment [23]. Similar results were reported by Izquierdo et al., who described two cases of DMG, in which an increase in plasma ct-DNA correlated with and preceded a shorter time interval to progression. On the other hand, they also reported two cases of hemispheric HGG with BRAFV600 mutations who showed an early decrease in plasma ct-DNA concentration, correlating with increased progression-free and overall survival (OS) [14].

Liquid biopsy, mostly from CSF but also from plasma, in DMG can successfully characterize H3K27mutations and predict tumor progression before it is radiographically detected. Moreover, liquid biopsy has the potential to distinguish between pseudo-progression and actual progression.

### 3.3. Medulloblastoma

Four studies analyzed the efficacy and utility of liquid biopsies in MB. Three studies (30%) included in this systematic review focused on MB only, whereas the study by Pagès et al. included MB patients (*n* = 27, 10.4%) in addition to other CNS tumors. The most common subgroup of MB in the included studies was group 4 (*n* = 75, 45%), followed by SHH (*n* = 42, 25%), WNT (*n* = 25, 15%) and group 3 (*n* = 23, 14%). Pagès et al. did not mention the different subgroups of MB [13,16,19,22]. The detection rate of cf-DNA in MB ranged from 26–64% [13,16] (Table 2). The study by Liu et al. detected the following mutations: CTNNB1, SUFU, KMT2D, CREBBP, KBTBD4, PT53, DDX3X, PTCH1 and KDM6A [13]. Sun et al. additionally identified KMT2C, SMARCA4, BCOR, EP300, NF1, SETD2, MED12 and SPEN as genetic alterations in MB patients [16]. All studies aimed to prove the feasibility of liquid biopsy in MB but also aimed to monitor disease progression with ct-DNA in MB patients [13,16,22] (Table 2).

The study by Liu et al. is the largest study on liquid biopsy in MB. They measured tumor-associated chromosomal copy-number variations (CNVs) as a surrogate of measurable residual disease (MRD). In 62% of cytologic-negative CSF samples, ct-DNA CNVs could be detected showing the superiority of liquid biopsy in comparison to traditional cytology. They showed that MRD correlated with the extent of the disease as well as prediction of progression. Patients who showed a disease progression had a significantly higher MRD during therapy than patients who showed stable disease. Furthermore, in 50% of all patients who achieved a radiographic response, MRD detection preceded a radiologically detected recurrence by over 3 months. Liu et al. could also show that detected MRDs in patients after radio- or during chemotherapy were associated with a significantly worse PFS [13].

The study by Li et al. investigated the different methylation patterns to characterize MB cf-DNA [22]. They detected reliable methylation signatures of ct-DNA in CSF. Moreover, in one patient the methylation signature changed in correlation with treatment response in serial CSF samples, which allows for monitoring disease progression [22].

Sun et al. investigated in how many patients the CSF obtained cf-DNA and genomic DNA from tumor tissue share alterations and they found that in only 22% shared alterations were identified, indicating a high intratumoral change in mutations throughout the disease. All these patients had in common that their CSF sample was taken intraoperatively compared to the remaining patients where CSF samples were obtained later [16]. High amounts of cf-DNA correlated with metastasized or progressive disease in 6 patients (10%) [16].

MB has a high rate of tumor mutation throughout the course of the disease. Ct-DNA in the CSF can be used as a surrogate marker of measurable residual disease and correlates with response to therapy and progression of the tumor up to three months before radiographic detection of tumor progression.

### 3.4. Other Pediatric CNS Tumors

Only one study included various types of CNS tumors, including low grade gliomas (*n* = 102), atypical teratoid rhabdoid tumors (ATRT, *n* = 5), ependymomas (*n* = 2), pineoblastomas (*n* = 1) and others, including craniopharyngioma, germ cell tumors, hemangioblastoma, meningioma, nerve sheath tumor, choroid plexus tumors and pineal tumors (*n* = 7) [19]. In nearly all samples, tumor fraction was <1% in cf-DNA, which limits the power of detection, and most of the tumors showed low rates of mutations [19].

Some of the other studies also included different types of tumor but only as negative controls or subanalysis and not in a systematic manner [13,21]. In this setting, however, Liu et al. also showed that their findings regarding MB are applicable to other embryonal tumors such as ATRT [13].

Low-grade tumors express a low rate of ct-DNA, making their characterization more cumbersome.

## 4. Discussion

To our knowledge, this is the first systematic review regarding the role of liquid biopsy in pediatric brain tumors. Based on this systematic review, CSF has the highest level of ct-DNA in comparison to blood or urine and is the preferred corporeal fluid for liquid biopsy in pediatric brain tumors. Liquid biopsy results showed successful monitoring and prediction of disease progression in DMG and MB [13,14,15,16,19,20,21,22,23].

### 4.1. Tumor Detection and Characterization with Liquid Biopsy

Positive detection rates of ct-DNA in CSF range from 26–96% in high-grade tumors but in a mixed cohort ct-DNA levels were extremely low, limiting standardized use of liquid biopsy [13,14,15,16,19,21,23]. Moreover, ct-DNA represents only a small percentage of overall cf-DNA that is produced by cell turnover. Harvesting enough of this small fraction of ct-DNA with a short half-life of around 2 h remains one of the main challenges in liquid biopsy [11,24]. Other parts of the liquid biome are micro-RNA and proteins; however, due to the single-strand nature of RNA, its analysis is more challenging than of ct-DNA [11]. Several studies have shown that ct-DNA is increased in patients with tumors adjacent to the ventricular system and even different gradients of ct-DNA in the ventricular system in relation to the tumor location were identified [13,23]. The much lower ct-DNA fraction in blood compared with CFS is probably due to the blood–webrain barrier (BBB), which makes liquid biopsy more difficult in CNS malignancies compared to other organ system tumors [25,26]. The BBB is partially disrupted in high-grade glioma although to a lesser extent than assumed, but it is widely accepted that the BBB is disrupted by radio- and chemotherapy, which could lead to a distribution of ct-DNA into the bloodstream and explain the higher detection rate after initiation of therapy [14,15,23,27]. This could explain why the study by Panditharatna et al. detected a high fraction of ct-DNA in plasma, allowing them to monitor the response to treatment [15]. Moreover, as ct-DNA stems mainly from necrotic cells, it is assumed that with increased tumor-cell turnover, induced by rapid growth or metastasis, the release of ct-DNA will increase [15,28,29]. These assumptions also account for the lower ct-DNA fraction detected in low-grade tumors, due to their slower growth. In addition, low-grade tumors often express only single mutations in their DNA, whereas high-grade tumors can harbor multiple ones, facilitating their detection via liquid biopsy [19,30,31]. Some studies in adult MB show higher detection rates compared to the pediatric studies, which is probably due to the higher rate of mutations in adult tumors compared to pediatric MB [22,32]. Hence, in low-grade tumors an increase in the detection rate could be reached by harvesting more biological fluid; however, in clinical practice, this can be cumbersome, especially in children where the collection of high volumes of blood or CSF might not be safe [19]. In the included articles, only few mutations are described for DMG or HGG, whereas various are reported for MB. Moreover, the study by Sun et al. showed that only 22% of genetic alterations were similar in the obtained CSF and tumor tissue and they found more mutation in the CSF samples than in the tumor itself [16]. These differences could be accounted for by biopsy sampling errors, which are a known problem in conventional biopsy of a heterogenous tumor. Moreover, MB and HGG, albeit to a lesser extent, are known to change their molecular and methylation profile over time, throughout therapy and during disease progression [16,33,34,35].

### 4.2. Disease Monitoring with Liquid Biopsy

Several studies have shown that liquid biopsy is feasible and reliable in DMG and MB to detect and monitor disease progression [13,14,15,16]. This is especially useful in highly eloquent tumors such as DMG, in which biopsies are associated with a relatively high morbidity [36]. Against the traditional practice not to biopsy brainstem tumors suggestive of DMG, nowadays a tissue diagnosis is sometimes helpful to guide molecular based therapy and especially inclusion into clinical studies [14,37,38]. Furthermore, the study by Cantor et al. could detect spikes in ct-DNA up to three months prior to radiographic progression. Similar results were detected for MB [13]. High ct-DNA after treatment correlated with a short PFS and an increase in ct-DNA in MB also preceded radiographic progression in half of all tested patients [13]. Particularly in high-grade tumors, such as DMG, with a dismal prognosis with short OS, early detection of disease progression can be of paramount importance to guide therapy. However, this would mean that patients would need to undergo regular sampling of CSF at the time, which can be unpleasant or could even predispose them to CSF infections [23,39,40]. However, as liquid biopsy is becoming more widespread, extraction rates will probably improve and regular blood sampling could be sufficient for tumor monitoring. Liquid biopsy cannot only detect early tumor progression but was also shown to distinguish between pseudo-progression and progression [23]. Despite all technical improvements in neuroimaging, it remains challenging to clearly distinguish between tumor progression or pseudo-progression caused by a reaction of the tumor to the therapy [41]. However, larger clinical trials will be needed to confirm these findings and standardize liquid-biopsy-centered tumor monitoring.

### 4.3. Future Prospective of Liquid Biopsy in PBT

Currently, liquid biopsy in PBTs is mainly used in the setting of clinical studies and not as a standard clinic diagnostic platform yet [14,20,21,22]. This is mostly due to the low ct-DNA extraction rates and low numbers of patients [14,15,19]. Most studies included in this review still use tumor tissue as a positive quality control and have not yet been generally established to replace biopsy for tumor diagnosis. However, liquid biopsy in CSF and blood reliably detects H3K27M mutations, which allows tumor diagnosis and monitoring of DMG [20,23]. Similar results are available for pediatric MB, which can be successfully detected and monitored with liquid biopsy [13,16]. Preferably, liquid biopsy could replace biopsy for any PBT, which would be especially useful in incidentalomas [19,42,43]. So far, however, ct-DNA extraction has proven to be difficult in low-grade PBT and no standardized or reliable measurements for PBTs in general are available yet [19]. So far, the clinical use of liquid biopsy seems to be restricted to high-grade PBTs, but, with the technical advances made in DNA extraction and purifying techniques, liquid biopsy might become the standard tool for systematic screening and diagnosis of PBT.

### 4.4. Limitations

Despite conducting a systematic review, this study has several limitations. First, we searched only two databases and only included studies in English. Further, we focused our search on pediatric tumors, which could lead to omitting certain studies that included both adult and pediatric patients. Moreover, this study contains inherent publication bias due to possible negative unpublished trials. Based on the available data, even if we had included a broader spectrum of papers (of which, most would be phase one trials or case reports), conducting a meta-analysis was not feasible at this point. In addition, most of the included studies had small numbers of patients and ct-DNA extraction rates were low, resulting in small numbers for statistical analysis with an increased risk of skewed results.

## 5. Conclusions

This systematic review shows that, to date, liquid biopsy is primarily useful in high-grade pediatric brain tumors such as diffuse midline glioma or medulloblastoma. CSF as substrate for ct-DNA extraction is superior to blood or urine. In both high-grade gliomas and medulloblastomas, liquid biopsy can successfully characterize and monitor tumor progression and can detect tumor progression prior to radiographic detectable disease progression. These results need to be confirmed and standardized in larger clinical trials in the future.

## Figures and Tables

**Table 1:** Overview of the included studies.

Author and Year of Publication	No of Patients	Analyzed Tumor	Analyzed Corporeal Fluids	Targeted Mutations	Method of Analysis	Results
Pagès et al. 2022 [19]	258	CNS tumors (102 LGG, 35 HGG, 27 MB, 5 ATRT, 2 ependymoma, 1 pineoblasotma, 7 others)	CSF, blood, urine	multiple	Ultra-low-pass whole genome sequencing (ULP-WGS)	Low levels of ct-DNA <1%,
Li et al. 2021 [20]	7	DMG	CSF, blood	H3K27M	Digital droplet PCR (ddPCR)	Reliable detection of H2K27M mutation with liquid biopsy
Panditharatna et al. 2018 [15]	48	DMG	CSF, blood, cyst fluid	H3K27M	ddPCR	Detection of response to radiotherapy and recurrence in plasma
Huang et al. 2017 [21]	11	DMG	CSF	H3K27, H3.3G34	Sanger sequencing for H3F3A and HIST1H3B, and Nested PCR with primers specific to H3F3A	Liquid biopsy successfully detects mutations in DMG
Izquierdo et al. 2021 [14]	33	DMG and HGG	CSF, blood, cyst fluid	H3K27, H3.3G34, BRAFV600E, G328V, IDH1, TP53, PIK3CA, MYCN	ddPCR	Successful detection of mutations in DMG
Cantor et al. 2022 [23]	28	DMG H3K27 positive	CSF, Blood	H3K27	ddPCR	Spike in ct-DNA detects and predicts recurrence prior to imaging, distinguishes between progression and pseudo-progression
Liu et al. 2021 [13]	123	MB	CSF	CTNNB1, SUFU, KMT2D, CREBBP, KBTBD4, PT53, DDX3X, PTCH1 KDM6A	Low coverage whole genome sequencing (lcWGS)	Ct-DNA higher in metastatic disease, increase with disease progression prior to radiological progression, higher ct-DNA levels in tumors close to CSF system
Sun et al. 2021 [16]	58	MB	CSF, blood	KMT2D, KMT2C, SMARCA4, BCOR, TP53, PTCH1, EP300, NF1, SETD2, MED12, SPEN	Next-generation sequencing (NGS)	Increase in ct-DNA in disease progression
Li et al. 2020 [22]	4	MB	CSF	Methylation	Whole-genome bisulfite sequencing (WGBS) and anti–cytosine-5-methylenesulfonate (anti-CMS) immunoprecipitation sequencing (CMS-IP–seq)	Detection of different methylation patterns, tumor-specific methylation pattern changes throughout therapy and indicates response, significant association with PFS

Abbreviations: PCR = polymerase chain reaction, CNS = central nervous system, ct-DNA = circulating tumor DNA, CSF = cerebrospinal fluid, LGG = low-grade glioma, HGG = high-grade glioma, MB = medulloblastoma, ATRT = atypical rhabdoid teratoid tumor, DMG = diffuse midline glioma.

**Table 2:** The role of liquid biopsy in different tumor types.

	Specific Mutations	Detection	Monitoring of Disease	Differentiation Progression vs. Pseudoprogression
**High-grade glioma (including DMG)**	H3K27, H3.3G34, BRAFV600E, G328V, IDH1, TP53, PIK3CA, MYCN	Reliable detection in CSF (64–96.5%)	Response to radiotherapy (plasma) (83%) and recurrence (plasma/CSF) (60%) measurable recurrence can be detected before radiographic recurrence (50%)	Differentiation between progression/pseudoprogression/ pseudoresponse (2 patients only, 18%)
**Medulloblastoma**	CTNNB1, SUFU, KMT2D/C CREBBP, KBTBD4, PT53, DDX3X, PTCH1 KDM6A SMARCA4, BCOR, EP300, NF1, SETD2, MED12, SPEN	Reliable detection in CSF (26–85%) 62% of cytology negative CSF samples were positive for ct-DNA	Response to treatment detectable progression can be detected before radiographic progression (50%) MRD during therapy correlates with shorter PFS	-

Abbreviations: DMG = diffuse midline glioma, CSF = cerebrospinal fluid, MRD = measurable residual disease.
